# Development of a richer measure of health outcomes incorporating the impacts of income inequality, ethnic diversity, and ICT development on health

**DOI:** 10.1186/s12992-018-0385-2

**Published:** 2018-07-20

**Authors:** Rezwanul Hasan Rana, Khorshed Alam, Jeff Gow

**Affiliations:** 1 School of Commerce, University of Southern Queensland, West, Street, Toowoomba, Qld 4350 Australia; 2 School of Accounting, Economics and Finance, University of KwaZulu-Natal, Durban, South Africa

**Keywords:** Health outcomes, Income inequality, Ethnic diversity, ICT development, Public health expenditure, SmartPLS, Panel data, OECD, I10, I31, H51, C33, C43

## Abstract

**Background:**

In the literature, measuring health outcomes usually entails examining one dependent variable using cross-sectional data. Using a combination of mortality and morbidity variables, this study developed a new, richer measure of health outcome. Using the health outcome index, this study investigated the impacts of income inequality, levels of ethnic diversity and information and communication technology (ICT) development on health using panel data.

**Methods:**

Partial least squares regression based on a structural equation model is used to construct a health outcome index for 30 OECD countries over the period of 2004 to 2015 using SmartPLS software. Then, panel corrected standard errors estimation and pooled ordinary least square regression with Driscoll and Kraay standard errors approaches were used to investigate the key determinants of health outcomes. Both methods are efficient when the panel data is heteroscedastic and the errors are cross-sectional dependent.

**Results:**

Income inequality, level of ethnic diversity and development in ICT access and use have an adverse effect on health outcomes, however, development in ICT skills has a significant positive impact. Moreover, OECD countries with a higher percentage of publicly funded healthcare showed better public health compared to countries where the percentage is smaller. Finally, rising incomes, development of technologies and tertiary education are key determinants for improving health outcomes.

**Conclusions:**

The results indicate that countries with higher levels of income inequality and more ethnically diverse populations have lower levels of health outcomes. Policymakers also need to recognise the adverse effect of ICT use on public health and the benefits of public healthcare expenditure.

**Electronic supplementary material:**

The online version of this article (10.1186/s12992-018-0385-2) contains supplementary material, which is available to authorized users.

## Background

The determinants of health outcomes include well-known factors such as income, medical technology and education. Some recent empirical studies have focused on the important role of information and communication technology (ICT) [1], income inequality [2], ethnic diversity [1, 3, 4] and public healthcare expenditure [5] to explain the observed health outcomes of countries. However, most previous research (both time-series and panel data estimation) have used a single variable, such as life expectancy or mortality rates (infant, maternal or under-five) to measure health outcomes [5–7]. Yet other studies have used outcome variables such as the prevalence or incidence of diseases or infections, the number of visits and readmissions to hospitals, hospital length of stay, work absences due to illness, perceived health status, quality-adjusted life years and disability-adjusted life years. Each of these measures and some recently developed health outcome indices (including the ‘better life index published’ by the Organisation for Economic Co-operation and Development (OECD) and ‘urban health index’ published by the World Health Organisation (WHO) is justifiable to answer individual research questions (often with cross-sectional data). Although reliable, these measures are inadequate for time series and panel data analysis. This is because continuous data on some of the indicators (e.g. self-reported health, disability, healthcare access) used in these indices are unavailable. For example, none of the health outcome indices provide data for an adequate number of time periods to enable statistically robust estimation to be undertaken. Also, studies estimating health outcomes with panel data are often obliged to use a single variable (mostly mortality variables) for estimation. Unfortunately, even for the OECD countries, which have rich sources of data for both mortality and morbidity variables, a comprehensive health outcome index suitable for panel data estimation is non-existent.

The current study constructs a health outcome index that incorporates both mortality (e.g. life expectancy and infant mortality) and morbidity (e.g. perceived health status) variables. The main objective of this study is to re-examine the factors that determined health outcomes for 30 OECD countries over the period 2004 to 2015. Along with some conventional determinants (e.g. income, education and technology), the estimated model also incorporates the influence of income inequality, ethnic diversity, development in ICT, and public health expenditure on health outcomes into the estimation process.

Past empirical findings have showed that societies with unequal incomes often experience poorer health outcomes [8–11]. However, the hypothesis that income inequality contributes to poorer health outcomes is not definitively determined with mixed empirical findings. Some studies have found a significant relationship between health outcomes (mortality, longevity) and income inequality [2, 8, 9, 12, 13] and others have concluded no significant association [11, 14, 15]. Furthermore, Rözer and Volker [16] found that the impact of income inequality on health reduces to zero after the age of 36 and Li and Zhu [10] reported a U shaped relationship, which means that at lower-levels of inequality, health outcomes improve with rising inequality and vice-versa. Hence, despite the large volume of literature, it is seemingly impossible to definitively determine the causality of income inequality on public health outcomes [17]. However, these past studies have mostly used cross-sectional data and a single variable to measure health outcomes. The health impacts of inequality will develop over a long period of time as the negative social consequences of that inequality will gradually take account of an individual’s health status [18]. Therefore, a representative health outcome index and panel data of 12 continuous years have been used to generate a better understanding of the issue.

Another key variable of interest in this study is ethnic diversity. Previous researchers have primarily focused on whether there are disparities in healthcare access, use and health status amongst majority and ethnic minority population [3, 4, 19, 20]. Most of the empirical findings indicated that ethnic minorities experience poorer access to healthcare services and their health status is significantly inferior to the majority of the population [3, 21]. Nonetheless, it is still unclear as to whether having higher levels of ethnic diversity reduces the overall health status of a country or not. Moreover, no study has yet examined the influence of public health expenditure on the potential adverse effects of higher levels of ethnic diversity on health outcomes.

Better health outcomes are also firmly linked with ICT development. ICT can support and promote efficient disease management and prevention [1], effective communication and reduction in the knowledge gap between patient and provider [22, 23], facilitate long-distance medical consultations [24], underpin the prevention of and supervision of public health risks [25, 26] and boost the performance and administration of the health system [27]. On the contrary, some empirical studies reported adverse effects of ICT on health outcomes. For example, Kim et al. [28] found that higher usage of the internet is associated with irregular food intake; Rosell et al. [29] indicated an association between mental distress and ICT use; Booth et al. [30] linked ICT use with less physical activity; and Punamäki et al. [31] concluded that ICT use is related to lower perceived health status. The contradictory findings may arise because previous studies used variables of choice that most suited the study purpose- to scale health outcome (mortality or morbidity) or ICT development (adoption or diffusion). Therefore, the overall impact of ICT developments on health outcomes is still unconfirmed [24]. In particular, what is the impact on overall public health when there is increased access to and use of ICT?

In light of the above discussion, the primary objective of this study is to examine the impact of income inequality, ethnic diversity and ICT development on health outcomes in the OECD countries utilising a large panel data. The secondary objective is to construct a health outcome index with indicators representing both mortality and morbidity variables. This study, therefore, investigates the following key questions: (i) does income inequality significantly affect health outcomes? (ii) do countries with greater levels of ethnic diversity have lower health status than countries with lower levels of diversity? (iii) what impact does ICT development (access, use and skill level) have on health outcomes? and (iv) does public health expenditure significantly improve health outcomes? These questions are empirically investigated by developing a health outcome index and using a panel dataset of OECD countries over the period of 2004 to 2015 (Additional file 1: Appendix A).

This study will make the following contributions to the existing literature. First, instead of using a single variable to measure health outcome, a health outcome index is constructed using both morbidity and mortality variables in a partial least square estimator based on a structural equation model (PLS-SEM). To the best of the authors’ knowledge this is the first study that has used perceived health status as an indicator of health outcome in panel data analysis. In the OECD database perceived health status was measured with the question “How is your health in general” and the responses were recorded in a Likert scale (from very bad to very good). Second, given that the adverse effects of income inequality on health occurs over time, this study assembled income inequality data and constructed a health outcome index over 12 continuous years. Third, the relationship between higher ethnic diversity and overall health outcomes was examined. Previous studies have investigated health care access of ethnic minorities but no previous study has examined health outcome variances across countries with different levels of ethnic diversity. Fourth, the relationship between ICT development and health outcome was re-examined using credible ICT development index data from the International Telecommunication Union (ITU). Furthermore, this study also investigated which aspect of ICT (access, use or skills) development is most strongly related to population health outcomes. Fifth, unlike previous studies, this study adopted a heterogeneous panel study method, rather than use homogenous panel data analysis. This enables control of cross-section dependence in the panel data by using OLS estimation with panel-corrected standard errors.

The paper consists of five sections. Section “Methods” outlines the model variables and research method. Section “Results” shows the results and section “Discussion and Policy Implications” consists of the discussion and finally, section “Conclusions” draws conclusions of the study.

## Methods

### Data and variables

This paper utilises a heterogeneous panel data for 30 OECD countries over the period 2004 to 2015. The rationale behind selecting the countries and the time period is the availability of data, especially for the variables of Gini coefficient and perceived health status. Data on infant mortality rate and income inequality came from World Development Indicators database [32]; ICT index data from World Telecommunication/ICT indicators database [33], population ethnicity data from the CIA World Factbook [34] and the remainder was sourced from the OECD database [35].

For the purpose of empirical analysis, apart from the measured variable HO*index*, other two key variables of interest are: income inequality (Gini) measured by the *Gini coefficient or Gini index* and the ICT*index* that represents the ICT development index which ranks all countries based on access, use and skills index [33]. To investigate the role of ethnic diversity (Eth*dum*) and public health expenditure (PHE*dum*), two dummy variables were generated. Ethnic diversity was low when the percentage of the largest ethnic group is high and vice versa. For example, in South Korea, 98% of the population are from the one ethnic background (Korean) yet in Switzerland, it is only 65%. Average ethnic diversity was calculated for the 30 countries (81.30%) and the variable was recorded 1 for countries below the average value and 0 otherwise. This being a time-invariant variable, only 2014 data was used. For PHE*dum* the mean value of public health expenditure as a percentage of total health expenditure was calculated. Countries with values above the average (71.91%) were scaled as 1 (generous levels of public health expenditure) and 0 otherwise. The average value in the year 2004 was 70.72 and 72.97% in 2015.

### Model specifications

The estimated model also controlled for the log of GDP per capita (lnGDPpc) expressed at USD current PPP prices; the percentage of obese population (males 15–64 years) (ObstM); the percentage of population (between 15 and 64 years) with tertiary education (TerEdu); public expenditure on research and development (RdExp) as a proxy for technological advancement; growth in actual hours of work per person per year (lnHrWrk); and the suicide rate (SudRt) as total suicides per 100,000 population as proxy for the overall mental health status of a country [36, 37]. Any missing data were replaced by linear interpolation [38]. The empirical models are specified as follows,

1 $$ \mathrm{HO}{index}_{it}=\alpha +\kern0.5em {\beta}_1\mathrm{ICT}{index}_{it}+{\beta}_2{\mathrm{Gini}}_{it}+{\beta}_3{\mathrm{lnGDPpc}}_{it}+{\beta}_4{ObsM}_{it}+{\beta}_5{TerEdu}_{it}+{\beta}_6\ {RdExp}_{it}+{\beta}_7\ {lnHrWrk}_{it}+{\beta}_8{SudRt}_{it}+{e}_i $$

2 $$ \mathrm{HO}{index}_{it}=\alpha +\kern0.5em {\beta}_1\mathrm{ICT}{index}_{it}+{\beta}_2{\mathrm{Gini}}_{it}+{\beta}_3{\mathrm{lnGDPpc}}_{it}+{\beta}_4{ObsM}_{it}+{\beta}_5{TerEdu}_{it}+{\beta}_6\ {RdExp}_{it}+{\beta}_7\ {lnHrWrk}_{it}+{\beta}_8{SudRt}_{it}+{\beta}_8{Dummy}_{it}+{e}_i $$

3 $$ \mathrm{HO}{index}_{it}=\alpha +\kern0.5em {\beta}_1\mathrm{ICT}{index}_{it}+{\beta}_2{\mathrm{Gini}}_{it}+{\beta}_3{\mathrm{lnGDPpc}}_{it}+{\beta}_4{ObsM}_{it}+{\beta}_5{TerEdu}_{it}+{\beta}_6\ {RdExp}_{it}+{\beta}_7\ {lnHrWrk}_{it}+{\beta}_8{SudRt}_{it}+{\beta}_8 Interaction\ {\mathit{\operatorname{var}}}_{it}+{e}_i $$

Lastly, some interaction variables were generated to better understand the relationship among the variables in the model and to test some additional hypothesis. They are:

$$ \mathrm{EthPhe}=\mathrm{Eth} dum\ \mathrm{multiplied}\ \mathrm{by}\ \mathrm{PHE} dum $$

$$ \mathrm{SklPhe}=\mathrm{ICT} skl\ \mathrm{multiplied}\ \mathrm{by}\ \mathrm{PHE} dum $$

Equations 1, 2 and 3 are used for statistical estimation by panel corrected standard errors (PCSE) and by pooled OLS Driscoll and Kraay standard errors (DKSE) models. Initial diagnostic tests showed that the panel data displayed cross-section dependence [39] and heteroskedasticity [40] but no serial correlation [41]. Ignoring these issues will seriously compromise the estimated results. The PCSE estimation method proposed by Beck and Katz [42] can account for these data difficulties and provide accurate estimates from linear models with panel data [43, 44]. Driscoll and Kraay [45] recommended a nonparametric covariance matrix estimator which can generate consistent standard errors in the presence of heteroscedasticity when the errors are spatial and temporal-dependent [46]. These estimation approaches will provide efficient and robust outcomes, suitable for formulating accurate conclusions.

### Construction of the health outcome index

The rationale behind constructing the health outcome index was the unavailability of data for the appropriate indicator variables with which the health outcome indices were developed. This study does not propose that the newly created index is better than the existing ones. However, it made panel estimation possible using a continuous time series dataset. The reliability of the index was tested by estimating the Cronbach coefficient alpha and the robustness and sensitivity of the index was examined by including and excluding indicators and altering weights in the aggregation process [47]. The findings are reported in Additional file 1: Appendix A.

The construction of the health index has four steps which include data of 36 countries for the year 2014.

#### Variable selection

The initial idea was to construct a health outcome index with three mortality variables (life expectancy at birth, infant mortality and death due to cancer) and three morbidity variables (perceived health status, absence at work due to illness and low birth weight). However, the partial least square based structural equation model (PLS-SEM) showed that only three variables; life expectancy at birth (LEB), infant mortality rate (IMR) and perceived health status as Bad (PHSB) were significant variables in the model.

To ensure that all three variables have a similar causal direction towards the dependent variable (i.e. health outcome data for the life expectancy at birth) LEB was modified into LEB1 (100- LEB) with the hypothesis that 100 years is the highest achievable life expectancy in the time period. Hence, a country with LEB = 82 had a revised LEB1 value of 18 (100–82) and a country with LEB 75 earned a LEB1 value of 25 (100–75). This ensured that all the indicator variables LEB1, IMR and PHSB are negatively related to health outcome, the latent variable. Additional file 1: Appendix A illustrates the detail process of the selection of the indicators.

#### Calculating weights

The estimation method for constructing the index is founded on the structural equation model (SEM) proposed by Wright [48]. The approach incorporates the advantages of both multiple regression and principal components analysis [49, 50]. Using SEM we have identified the structural model with the set of indicators (LEB1, IMR and PHSB) used as the latent variables. One benefit of using the SEM approach is that it can measure the reliability of the relationship between indicators and the latent variable, and is suitable to control for the multicollinearity problem among the indicators in the model. Urbach and Ahlemann [51] outlined the detailed methodology of the SEM approach.

This study used the PLS-SEM which aims to estimate the association between indicator variables and the latent variables with a system of autonomous equations based on simple and multiple regressions [52]. This study employed the reflective measurement model which considers the indicator variables as reflections of the latent variables and the path of causality runs from construct to the indicators [52, 53]. The reflective measure tests the reliability of the estimation while considering the indicator variables as a linear combination of its latent variable. The estimated model is specified as,

$$ {x}_{pq}={\lambda}_{pq}{\xi}_q+{\varepsilon}_{pq} $$

where *x*_*pq*_ indicates indicator variable, *ξ*_*q*_ is the latent variable, *λ*_*pq*_ represents the generic loading coefficients of the *p*-th indicator variable in the q block and *ε*_*pq*_ is the residual terms of the generic indicator variables [53]. See Chin [52] for a detail discussion of the method.

SmartPLS (version 2) software was used to run the model.

**Fig. 1:**
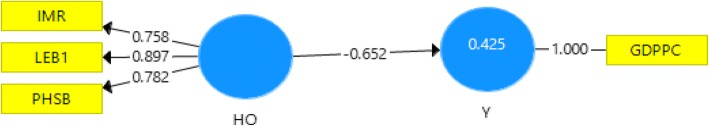
*p-values* of the proposed structural equation model

Lastly, the factor loadings for each indicator were divided by the summation of the factor loadings (0.758 + 0.897 + 0.782 = 2.437) of the three indicators to calculate the weights which are 0.311 (0.758/2.437) for IMR, 0.369 (0.897/2.437) for LEB1 and 0.320 (0.782/2.437) for PHSB (Fig. 1). This process normalised the corresponding loadings of each indicator and eased the comparability of the weights. All the variables are statistically significant at a 5% confidence interval and an R-Sq = 0.425 indicates that 42.5% of the observed variable (per capita GDP in Fig. 1) is explained by the indicators in the model.

#### Standardization of the indicators

The aim of this step was to accommodate disparities in the metrics and scales of the indicators. The score of each unit was standardised by dividing the difference between the current value and lowest value with the difference between the highest value and lowest value. This method has been used in previous studies, for example in constructing the ‘Urban Health Index’ [54].The simple standardisation formula is as follows,

$$ {\mathrm{I}}^{\mathrm{SD}}=\frac{\mathrm{CV}-\mathrm{LV}\ }{\mathrm{HV}-\mathrm{LV}} $$

where *I*^*SD*^ stands for the standardised value of the indicator, *CV, LV* and *HV* are the current value, lowest value and highest value, respectively, for each of the indicators among the 36 countries in a particular year.

#### Estimation of index scores

After standardisation of the values of the indicators, the next step was to integrate the *I*^*SD*^into a single composite index. To do so, standard value of each indicator was multiplied by corresponding normalised weights. Afterwards, each sub-index scores were summed for individual countries for each year to construct the health index scores. The basic health outcome index is described by the following equation,

$$ \mathrm{HO} index=\sum \left(\mathrm{ZLEB}1\ \mathrm{x}\ {\mathrm{W}}_{LEB1\kern0.5em }+\mathrm{ZIMR}\ \mathrm{x}\ {\mathrm{W}}_{IMR}+\mathrm{ZPHSB}\ \mathrm{x}\ {\mathrm{W}}_{PHSB}\right) $$

Here Z indicates the standardised value of the indicators. As mentioned, all of the indicators are negatively related to health outcome or better health status. Therefore, for ease of interpretation of the findings, we deducted the HO*index* scores from the value of 1 (the maximum attainable value). The modified value indicates that higher the index score, the better the health outcomes or health status of the country. Table 1 illustrates the HO*index* scores for the OECD countries for the year 2014.

**Table 1:** The calculated health outcome index and indicator variables for 2014

Ranking	Country	HO Index	LEB1	IMR	PHSB
1	Sweden	0.920882	17.7	2.4	4
2	Switzerland	0.906609	16.7	3.5	3.8
3	Iceland	0.901149	17.1	1.6	6.3
4	Spain	0.851465	16.7	3.6	8.3
5	Norway	0.850066	17.8	2.2	6.9
6	Ireland	0.847285	17.9	3.1	4
7	Canada	0.846275	17.9	4.4	3.1
8	Finland	0.830299	18.7	2	6.4
9	Netherlands	0.824627	18.2	3.3	5.4
10	Luxembourg	0.811795	17.7	1.6	8.3
11	France	0.806762	17.2	3.6	8.4
12	Italy	0.772004	16.8	3	12
13	Austria	0.764056	18.4	3	8.9
14	Germany	0.759641	18.8	3.2	8
15	Slovenia	0.744356	18.8	2.2	11
16	Belgium	0.736288	18.6	3.4	9.2
17	Denmark	0.728947	19.2	3	7.4
18	UK	0.723706	18.6	3.7	9
19	Greece	0.697221	18.5	3.7	10.8
20	USA	0.682182	21.2	5.7	2.8
21	Israel	0.648615	17.8	3.3	15.5
22	Korea	0.643678	17.8	3	15.9
23	Czech Republic	0.617804	21.1	2.9	11.6
24	Portugal	0.55644	18.8	3	18.3
25	Poland	0.465052	22.3	4.5	13.7
26	Estonia	0.436804	22.8	2.5	16.5
27	Slovak Republic	0.399524	23.1	6.1	12.7
28	Hungary	0.335858	24.1	5.3	15.9
29	Turkey	0.285885	22	12.3	11.8
30	Latvia	0.268991	25.7	7.2	17.1

Note: These 30 countries were selected due to availability of continuous time-series data

## Results

Table 2 shows the descriptive statistics of the variables. It includes the mean values and the range (highest minus lowest) of the values. During the study period the mean health outcome index improved slightly from 0.70 to 0.73 however, the disparity in health outcomes amongst the 30 countries reduced significantly from 0.77 in 2004 to 0.54 in 2015. This finding indicates that countries with historically poor health status are gradually closing the gap to countries with traditional better health status. Average income inequality reduced slightly (33.01 to 31.80) and the ICT development index increased from 4.55 to 7.70. Noticeably, the number of obese population (male) has risen alarmingly from 17.09 in 2004 to 22.43 in 2015 and the suicide rate showed a minor decrease from 114.12 to 111.67.

**Table 2:** Descriptive analysis

Variable	Mean	Max-Min	Mean	Max-Min	Mean	Max-Min	Mean	Max-Min	Mean	Max-Min	Mean	Max-Min
Year	2004		2005		2006		2007		2008		2009	
HO*index*	0.70	0.77	0.70	0.74	0.69	0.74	0.69	0.73	0.69	0.70	0.67	0.77
Gini	33.01	116.31	332.72	117.07	332.30	118.12	332.26	117.38	332.04	117.78	331.97	117.37
ICT*index*	44.55	33.64	55.29	44.90	55.67	44.32	66.05	33.74	66.43	33.95	66.60	44.03
ICT*acs*	55.58	55.22	66.10	44.89	66.61	44.57	77.13	44.24	77.02	33.98	77.20	33.93
ICT*use*	11.59	22.84	22.32	33.55	33.05	44.26	33.79	44.97	44.46	55.33	55.01	33.98
ICT*skl*	88.32	33.07	88.43	33.17	88.54	33.31	88.64	33.60	88.71	33.00	88.76	33.05
RdExp	11.69	33.48	11.73	33.56	11.75	33.67	11.76	33.98	11.87	33.88	11.92	33.68
lnGDPpc	110.17	11.78	110.22	11.76	110.31	11.75	110.37	11.74	110.42	11.69	110.38	11.68
ObstM	117.09	222.20	117.57	223.00	118.08	223.60	118.60	224.20	119.11	224.80	119.63	225.30
TerEdu	330.81	441.72	332.14	441.18	333.14	441.16	334.06	441.47	335.37	442.39	336.92	446.45
lnHrWk	77.47	00.52	77.47	00.51	77.46	00.50	77.46	00.48	77.46	00.45	77.45	00.46
SucdRt	114.12	226.20	114.04	227.70	113.41	227.90	112.83	224.10	112.72	226.70	112.77	227.00
Year	2010		2011		2012		2013		2014		2015	
HO*index*	0.68	0.77	0.65	0.74	0.66	0.78	0.67	0.63	0.68	0.63	0.73	0.54
Gini	331.91	117.56	331.93	117.13	332.11	116.14	332.10	115.47	331.78	115.47	331.80	115.60
ICT*index*	66.78	44.28	66.97	44.18	77.43	33.69	77.57	33.57	77.64	33.57	77.70	33.14
ICT*acs*	77.39	33.94	77.36	33.71	77.49	33.82	88.09	33.63	88.11	33.63	88.13	33.49
ICT*use*	55.56	44.01	55.67	55.87	66.07	55.62	66.36	55.47	66.60	55.47	66.84	55.06
ICT*skl*	88.81	33.10	88.81	33.07	88.83	22.63	88.93	22.82	88.92	22.82	88.92	22.63
RdExp	11.90	33.34	11.99	33.35	22.05	33.50	22.00	33.54	22.02	33.54	22.08	33.63
lnGDPpc	110.42	11.60	110.47	11.55	110.49	11.49	110.53	11.45	110.56	11.45	110.60	11.45
ObstM	220.09	225.80	220.55	226.10	221.02	226.40	221.50	226.80	221.95	226.80	222.43	227.60
TerEdu	337.76	447.57	338.58	444.95	339.92	444.69	441.00	444.68	441.80	444.68	442.05	443.82
lnHrWk	77.45	00.44	77.45	00.43	77.44	00.43	77.44	00.43	77.43	00.43	77.43	00.42
SucdRt	113.06	331.40	112.52	331.80	112.68	331.20	112.62	226.50	112.02	226.50	111.67	117.40

HO*index* Health outcome index, *Gini* Gini index, ICT*index* Information and communication technology, *acs* Access, skl Skil, *RdExp* Public expenditure on research and development, *lnGDPpc* Log of per capita gross domestic product, *ObstM* The percentage of obese population (males 15–64 years), *TerEdu* Percentage of total population (15 to 64) are tertiary educated, *lnHrWrk* Growth in actual hours of work per person per year, *SudRt* Suicide rate as total suicide per 100,000 population

Table 3 illustrates the findings of eq. 1 with four different models estimated by PCSE and Pooled OLS with DKSE approaches. For model 1, we used variable ICT*index;* for model 2 ICT*acs*; for model 3 ICT*use;* and for model 4 ICT*skl*. According to the PCSE approach, ICT development, income inequality, male obesity, growth in hours of work and the suicide rate have a negative impact on health outcomes in model 1. However, income inequality and the suicide rate are not significant. A one unit increase in the ICT development index reduces the health outcome index by 0.051. To further investigate the impact of ICT development, the variable was segregated into three parts. Notably, the findings of models 2, 3 and 4 indicated that although ICT*acs* and ICT*use* are inversely related to health outcome, conversely, improvement in ICT*skl* has a positive and significant effect on health. Moreover, expectedly, growth in income, technological development (R&D expenditure) and a rising percentage of tertiary educated population have a significant and positive influence on health outcomes. A 1% increase in per capita GDP increases the health outcome index by 0.328.

**Table 3:** OLS estimation results for models 1 to 4

Test	Pooled OLS DKSE				OLS PCSE			
Dep Var. (Health outcome)	Model 1	Model 2	Model 3	Model 4	Model 1	Model 2	Model 3	Model 4
ICT*index*	−0.051*** (0.002)				−0.027** (0.007)			
ICT*acs*		−0.033* (0.017)				−0.014* (0.008)		
ICT*use*			−0.039*** (0.003)				−0.024*** (0.004)	
ICT*skl*				0.063*** (0.013)				0.022** (0.012)
Gini	−0.012*** (0.009)	−0.010*** (0.001)	− 0.013*** (0.001)	−0.004*** (0.001)	− 0.002 (0.002)	−0.001 (0.002)	− 0.003* (0.002)	−0.000 (0.001)
*RdExp*	0.042*** (0.003)	0.037*** (0.002)	0.041*** (0.006)	0.016*** (0.002)	0.024** (0.011)	0.019* (0.011)	0.0 7** (0.010)	0.014 (0.011)
lnGDPpc	0.371*** (0.042)	0.365*** (0.056)	0.372*** (0.034)	0.342*** (0.016)	0.328*** (0.014)	0.311*** (0.043)	0.344*** (0.039)	0.282*** (0.037)
ObstM	−0.007*** (0.001)	− 0.009*** (0.002)	− 0.005*** (0.000)	− 0.011 (0.002)	− 0.007*** (0.002)	−0.009*** (0.002)	− 0.005*** (0.002)	−0.010*** (0.002)
TerEdu	0.004*** (0.001)	0.003*** (0.001)	0.005*** (0.001)	0.0002 (0.001)	0.002** (0.001)	0.001 (0.001)	0.002*** (0.001)	0.000 (0.001)
lnHrWrk	0.065 (0.026)	0.033 (0.029)	0.075 (0.025)	−0.062* (0.030)	−0.167* (0087)	− 0.196** (0.090)	−0.108 (0.078)	− 0.226** (0.090)
SudRt	−0.011*** (0.002)	− 0.011*** (0.001)	−0.011*** (0.002)	− 0.010*** (0.001)	−0.002 (0.002)	− 0.002 (0.002)	−0.004** (0.002)	− 0.002 (0.002)
*Obs*	339	339	339	339	339	339	339	339
*N-group*	30	30	30	30	30	30	30	30
*R-Sq*	0.7085	.6805	0.7488	0.6995	0.8594	0.8433	0.8518	0.8438
*VIF*_*mean*_					2.23	2.40	2.07	2.04
*CSD*_*p-value*_					0.000	0.000	0.000	0.000
*Hetero*_*p-value*_					0.000	0.000	0.000	0.000
*AutoCorr*_*p-value*_					0..17	0.15	0.19	0.13
*JB test*_*p-value*_					0.054	0.190	0.143	0.285

Notes: Significant at 10, 5, and 1% level is defined by *, **, and ***, respectively. Standard error appear in the parentheses. Obs indicates the number of observations, VIF shows the variance inflation factor test results for multicollinearity, Hetero is the test of heteroskedasticity, Autcorr is the first-order serial correlation test [41], CSD is the cross-section dependence test [37] and JB test is the Jarque-Bera residual normality test [55]

The results of the Pooled OLS DKSE estimation indicate similar signs for almost all the coefficients, however, there are some notable differences. The variable income inequality is not only negatively related to health outcome but is also significant. Growth in hours of work is positive but not significant and the suicide rate significantly impacts health outcomes. The only variable that has a different sign is growth in hours of work but it is not significant. For model 1, a one unit increase in income inequality and the suicide rate reduces the health outcome index by 0.012 and 0.011, respectively.

The diagnostic test results showed that the panel dataset exhibits heterogeneity and the errors are cross-sectional dependent. Serial correlation and multicollinearity were not detected and the errors are normally distributed [55]. All the results were significant at 95% confidence interval.

Table 4 shows three alternative versions of model 1 (with Eth*dum*, PHE*dum* and EthPhe) and one alternative of model 4 (with SklPhe). As expected, all the coefficient signs and significance level are similar to the previously estimated results in Table 3. However, there were minor changes in the coefficient values. The PCSE estimation results show that countries with higher ethnic diversity have lower health outcomes than countries with lower ethnic diversity and the result is significant at 1% confidence interval. In addition, countries where health expenditure is generously funded by government enjoy significantly better health outcomes. Moreover, countries with higher ethnic diversity and more generous public health expenditure are significant at 1%. Finally, countries with a higher ICT*skl* index and generous public health expenditure illustrates significantly higher health index values. The Pooled OLS DKSE approach also shows identical results.

**Table 4:** OLS estimation results with dummy and interaction variables

Test	Pooled OLS DKSE				OLS PCSE			
Dep var. (Health outcome)	Ethnicity*dum*	Public Health Exp*dum*	Interatction term 1	Interaction term 2	Ethnicity*dum*	Public Health Exp*dum*	Interatction term 1	Interaction term 2
ICT*index*	−0.057*** (0.007)	−0.054*** (0.007)	−0.052*** (0.007)		−0.031*** (0.007)	−0.030*** (0.006)	− 0.31*** (0.006)	
ICT*skl*				0.054*** (0.013)				0.012* (0.012)
Gini	−0.008*** (0.001)	−0.008*** (0.001)	− 0.007*** (0.001)	−0.002 (0.001)	− 0.001 (0.002)	−0.0001 (0.002)	− 0.0007 (0.002)	−0.002 (0.002)
RdExp	0.040*** (0.003)	0.046*** (0.004)	0.031*** (0.003)	0.019*** (0.002)	0.025** (0.011)	0.031*** (0.011)	0.019** (0.010)	0.019** (0.011)
lnGDPpc	0.373*** (0.038)	0.351*** (0.0445)	0.366*** (0.039)	0.330*** (0.018)	0.332*** (0.039)	0.314*** (0.042)	0.334*** (0.038)	0.265*** (0.038)
ObstM	−0.005*** (0.001)	−0.004*** (0.001)	−0.006*** (0.001)	− 0.009*** (0.002)	−0.005** (0.002)	− 0.004** (0.027)	−0.006*** (0.002)	− 0.008*** (0.002)
TerEdu	0.005*** (0.001)	0.004*** (0.001)	0.005*** (0.001)	0.000 (0.001)	0.003*** 0.001)	0.002** (0.001)	0.003*** (0.001)	0.001 (0.001)
lnHrWrk	0.008 (0.025)	0.207*** (0.028)	0.118*** (0.023)	0.063** (0.026)	−0.196 (0.085)	−0.012 (0.097)	− 0.061 (0.081)	−0.083 (0.102)
SudRt	−0.009*** (0.002)	−0.009*** (0.001)	− 0.009*** (0.001)	−0.009*** (0.001)	− 0.002 (0.002)	−0.002 (0.002)	− 0.002 (0.001)	−0.002 (0.002)
Eth*dum*	−0.076*** (0.011)				- 0.077*** (0.020)			
PHE*dum*		0.084*** (0.013)				0.085*** (0.026)		
EthPhe			−0.044*** (0.013)				− 0.054*** (0.018)	
SklPhe				0.008*** (0.001)				0.009*** (0.003)
*N-group*	339	339	339		339	339	339	
*Obs*	30	30	30		30	30	30	
*R-Sq*	0.7375	0.7296	0.7423		0.8566	0.8559	0.8599	

Notes: Significant at 10, 5, and 1% level is defined by *, **, and ***, respectively. Standard error appear in the parentheses. Obs indicates the number of observations

## Discussion

All estimated coefficients have the a priori anticipated signs. This confirms the validity of the health outcome index constructed in this study. Thus the method was then used investigate the key determinants of health outcome using longitudinal data while controlling for heteroskedasticity and cross-section dependence.

The results of the Pooled OLS with DKSE and PCSE estimation showed that income inequality negatively impacts health outcome for the OECD countries. However, this outcome is significant only in the Pooled OLS test. One observation is that to establish the relationship beyond doubt, maybe, time series data on a longer time scale is required [56] as income inequality often affects health through other components such as harmful health behaviour [57] and stress-related diseases [58]. Nonetheless, this study supports the income inequality hypothesis that the more unequal the society, the higher the probability of poorer health controlling for per capita GDP. Moreover, past empirical works often produced mixed conclusions, where some researchers supported this hypothesis [2, 8, 9, 13, 59] and others refuted it [15, 60].

In recent years, many organisations and governments have promoted the use and access of ICT in the health sector [24]. However, the findings of this study suggested that increasing ICT access and use in society has an adverse effect on health outcomes. A number of previous studies have discussed the potential health benefits of ICT development, access and use. Their findings concluded that ICT improves the diffusion and communication of health information and knowledge, therefore, facilitates increased health literacy and healthier lifestyles [26, 61]. As a result, increasing access and use of ICT can improve a country’s public health outcomes [62, 63]. Conversely, Punamäki et al. [31] found that ICT use is connected with lower perceived health status and yet others concluded that excessive usage of ICT can cause musculoskeletal problems [64, 65]. Other, empirical findings also associated ICT access and use with unhealthy lifestyles and obesity [28], mental health disorders such as lack of sleep and depression [58] and development of health compromising-behaviour in both men and women [66]. Therefore, ICT use and access have both positive and negative impacts on health. On balance, the results of this study show that the negative impacts outweigh the benefits. Not surprisingly, improvement in ICT skills (measured by the literacy rate) has been found to promote better health.

The level of ethnic diversity in most OECD countries is relatively low and the study findings show that countries with lower levels of diversity enjoy higher health status. This conclusion is consistent with previous empirical works which showed that ethnic minorities are subject to unequal access to health care services, and poorer health status [3, 4, 19, 64, 67, 68]. Nevertheless, the detrimental consequences of higher ethnic diversity on health outcome can be compensated in part by devoting a larger share of publicly funded health care expenditure to minority groups.

A common point of argument among researchers and policymakers is the utility of public welfare expenditure, especially in the healthcare sector. In this context, the present study provides strong evidence that where health care is generously financed by government better health outcomes are evidenced. The study results support previous findings of [5, 69–71] and contradicts Self and Grabowski [72] who found no such relationship in developed countries.

Finally, growth in per capita GDP, advancement in technology and increasing levels of tertiary education are pivotal components of better health outcomes in OECD countries. On the contrary, obesity plays a major role in deteriorating health status. The percentage of the obese population (male) has increased by 31% (authors own calculation) over the period 2004 to 2015, which can be a considerable cause of concern for future public health policies in these countries.

Some policy implications can be derived from the findings of this study.

First, this study examined the relationship between the development of ICT access, use and skills with overall public health outcomes. This study supports the hypothesis that overuse of ICT (by the general population) can have a negative impact on public health. Governments should create public awareness regarding the ill-effects of ICT use on health and recommend necessary actions to avoid future health problems.

Second, policymakers should recognise that having a population of greater ethnic diversity makes it more challenging to promote better health outcomes for all. This may be part of the answer to the puzzle as to why some countries could achieve better health outcomes with lower per capita expenditure on health. Populous countries such as the USA with higher levels of ethnic diversity may find it difficult to achieve the health status of Japan (a country with lower ethnic diversity). Again, policymakers should realise that improving health care access for ethnic minorities with publicly funded health care can improve the overall health of a country.

Third, understanding the effectiveness of public health expenditure in producing a healthy nation is very important. The findings of the study may be an important indication for governments especially those with comparatively lower health status. Even with the burden of rising health expenditure, governments should continue to fund the health system adequately.

Last, the issue of rising obesity in OECD countries should be urgently addressed. Governments must actively promote healthy lifestyles and discourage or control social and individual practices that cause obesity.

## Conclusions

A number of empirical studies have examined the factors affecting health outcome in OECD countries. These studies either used cross-section data or a singular variable to measure health outcome. This study constructed a health outcome index with indicators comprising both mortality and morbidity data for 30 OECD countries over the period of 2004 to 2015. The index represented overall health status in individual countries. Development of the index gave the opportunity to investigate the impact of income inequality, ethnic diversity and ICT development on health with a panel dataset. In addition, the estimated method controlled for heterogeneity and cross-section dependence which improved the reliability of the estimated outcomes.

The study results indicate that income inequality, ethnic diversity and developments in ICT access and use have a significant negative relationship with health outcome in the OECD countries. On the other hand, improvements in ICT skills level and a higher levels of publicly funded health expenditure show statistically significant and positive associations with better health outcomes. Rising national incomes, developments in technology and higher education levels also improve a country’s health outcomes. On the contrary, rising obesity amongst the adult population has serious negative consequences for health.

There are some limitations of the study. The panel dataset is restricted to only 12 years due to unavailability of data for some of the variables such as perceived health status and income inequality. However, the time frame (2004–2015) is important because during this time ICT development accelerated rapidly (Table 2) and ethnic diversity increased in the OECD countries. In addition, use of more morbidity indicators would have increased the credibility of the health outcome index. The panel data has shown significant heterogeneity and cross-section dependence. The results of this study, therefore, should be interpreted with caution.

### Highlights

1. This study constructed a new health outcome index using both mortality and morbidity variables.
2. Income inequality and higher level of ethnic diversity negatively impacts health outcomes in OECD countries.
3. Improvement in ICT access and use negatively, and ICT skills positively effects health outcomes.
4. OECD countries with larger portion of publicly funded health care show better health outcome compared to other countries.

## Additional file

Additional file 1: The country list, variable definitions and additional statistical analysis [73–84]. (DOCX 126 kb)
