# Supplementary material for: Development of a richer measure of health outcomes incorporating the impacts of income inequality, ethnic diversity, and ICT development on health
Source: Global Health. 2018 Jul 20;14:72. doi: 10.1186/s12992-018-0385-2 (PMC6054722; doi:10.1186/s12992-018-0385-2)
Supplement: Supplementary file 1 — The country list, variable definitions and additional statistical analysis [73–84]. (DOCX 126 kb) [file 12992_2018_385_MOESM1_ESM.docx]

**Appendix A**

**Appendix A**

**1. Country list:**

Australia*, Austria, Belgium, Canada, Czech Republic, Chile*, Denmark, Estonia, Finland, France, Germany, Greece, Hungary, Iceland, Ireland, Israel, Italy, Japan*, South Korea, Latvia, Lithuania*, Luxembourg, Mexico*, Netherlands, New Zealand*, Norway, Poland, Portugal, Slovak Republic, Slovenia, Spain, Sweden, Switzerland, Turkey, UK, USA,

* indicates countries that were not considered in the final statistical analysis due to unavailability of continuous time-series data.

**2. Variable definitions:**

Health Outcome = Measured with health outcome index Prepared with life expectancy, infant mortality and perceived health status data using the software SMART partial least square (Smart PLS)

ICT*index* = ICT development index value created by International telecommunication union (ITU)

ICT*acs* = ICT Access index value created by International telecommunication union (ITU)

ICT*use* = ICT Use index value created by International telecommunication union (ITU)

ICT*skl* = ICT skill index value created by International telecommunication union (ITU)

Gini = Income inequality (gini coefficient value)

RdExp = Research and development expenditure as percentage of total government expenditure.

lnGDPpc = Growth in per capita GDP current dollar PPP

ObstM = Percentage of male (15 to 64) of total population are obese.

TerEdu = Percentage of total population (15 to 64) are tertiary educated.

SudRt = Suicide rate total, per 100000 populations

Eth*dum* = Ethnic diversity dummy.

PHE*dum* = Public health expenditure dummy

EthPhe = (Interaction variable) Ethdum x PHEdum

SklPhe = (Interaction variable) ICTSkl x PHEdum

Pop65R = 100- Percentage of population of 65 years old out of total population

**3. Reliability analysis**

**Table 5: Reliability statistics of the indicators included in the health outcome index**

| **Case Processing Summary** | | | | | | | | | **Reliability Statistics** | | | | | |  |
| --- | --- | --- | --- | --- | --- | --- | --- | --- | --- | --- | --- | --- | --- | --- | --- |
|  | | | | N | | % | | | Cronbach's Alpha | | | N of Items | | |  |
| Cases | | Valid | | 36 | | 97.3 | | |  | | |  | | |  |
|  |  | Excluded | | 1 | | 2.7 | | | 0.681 | | | 3 | | |  |
|  |  | Total | | 37 | | 100.0 | | |  | | |  | | |  |
| **Parametric Correlations test** | | | | | | | | | | | | | |  |  |
|  | | | | | LEB1 | | IMR | | | | PHSB | | |  |  |
| LEB1 | Pearson Correlation | | | | 1 | | .611^**^ | | | | .573^**^ | | |  |  |
|  | Sig. (2-tailed) | | | |  | | .000 | | | | .000 | | |  |  |
|  | N | | | | 36 | | 36 | | | | 36 | | |  |  |
| IMR | Pearson Correlation | | | | .611^**^ | | 1 | | | | .288 | | |  |  |
|  | Sig. (2-tailed) | | | | .000 | |  | | | | .089 | | |  |  |
|  | N | | | | 36 | | 36 | | | | 36 | | |  |  |
| PHSB | Pearson Correlation | | | | .573^**^ | | .288 | | | | 1 | | |  |  |
|  | Sig. (2-tailed) | | | | .000 | | .089 | | | |  | | |  |  |
|  | N | | | | 36 | | 36 | | | | 36 | | |  |  |
| **Non parametric Correlations tests** | | | | | | | | | | | | | | | |
|  | | | | | | | | LEB1 | | IMR | | | PHSB | | |
| Kendall's tau_b | | | LEB1 | Correlation Coefficient | | | | 1.000 | | .302^*^ | | | .342^**^ | | |
|  |  |  |  | Sig. (2-tailed) | | | | . | | .011 | | | .004 | | |
|  |  |  |  | N | | | | 36 | | 36 | | | 36 | | |
|  |  |  | IMR | Correlation Coefficient | | | | .302^*^ | | 1.000 | | | .092 | | |
|  |  |  |  | Sig. (2-tailed) | | | | .011 | | . | | | .436 | | |
|  |  |  |  | N | | | | 36 | | 36 | | | 36 | | |
|  |  |  | PHSB | Correlation Coefficient | | | | .342^**^ | | .092 | | | 1.000 | | |
|  |  |  |  | Sig. (2-tailed) | | | | .004 | | .436 | | | . | | |
|  |  |  |  | N | | | | 36 | | 36 | | | 36 | | |
| Spearman's rho | | | LEB1 | Correlation Coefficient | | | | 1.000 | | .446^**^ | | | .457^**^ | | |
|  |  |  |  | Sig. (2-tailed) | | | | . | | .006 | | | .005 | | |
|  |  |  |  | N | | | | 36 | | 36 | | | 36 | | |
|  |  |  | IMR | Correlation Coefficient | | | | .446^**^ | | 1.000 | | | .129 | | |
|  |  |  |  | Sig. (2-tailed) | | | | .006 | | . | | | .454 | | |
|  |  |  |  | N | | | | 36 | | 36 | | | 36 | | |
|  |  |  | PHSB | Correlation Coefficient | | | | .457^**^ | | .129 | | | 1.000 | | |
|  |  |  |  | Sig. (2-tailed) | | | | .005 | | .454 | | | . | | |
|  |  |  |  | N | | | | 36 | | 36 | | | 36 | | |
| *. Correlation is significant at the 0.05 level (2-tailed). | | | | | | | | | | | | | | | |
| **. Correlation is significant at the 0.01 level (2-tailed). | | | | | | | | | | | | | | | |

Table 5 shows the reliability statistics of the indicator variables which was measured with a widely used approach (Cronbach’s Alpha) developed by Cronbach [73]. Previous studies advocated different desirable values of ‘Alpha’ (0.70 to 0.95) [74, 75]. However, number of items used in the model influences the values of ‘Alpha’ significantly [76] and the value increases with the length of the test [77]. According to Pedhazur and Schmelkin [78] the ‘Alpha’ value 0.7 should not be used as a general guideline rather should be used from the outline of the research objectives. In addition several previous studies concluded that based on the context of the research ‘Alpha’ values as low as 0.5 [79] and 0.6 are acceptable [80]. Hence, the Cronbach’s Alpha value of approximately 0.7 with only 3 items is justifiable and validates the construct/ indicators used to measure health outcome in this study. Future studies with larger (available) dataset should use more items (strongly correlated to each other) for a better reliability statistics. Finally, the parametric and non-parametric correlation tests show that the indicators are significantly correlated to each other.

**4. The sensitivity and robustness of the health outcome index**

The stability of the constructed health outcome index was examined through inclusion and exclusion of indicators as well as altering the weights. The process and estimated outcomes are discussed below with Figure 2 and Table 6

**Figure 2 Sensitivity and robustness of the indicators (exclusion and inclusion of indicators)**

| **Outer/factor loading values** | **T-value** |
| --- | --- |
| 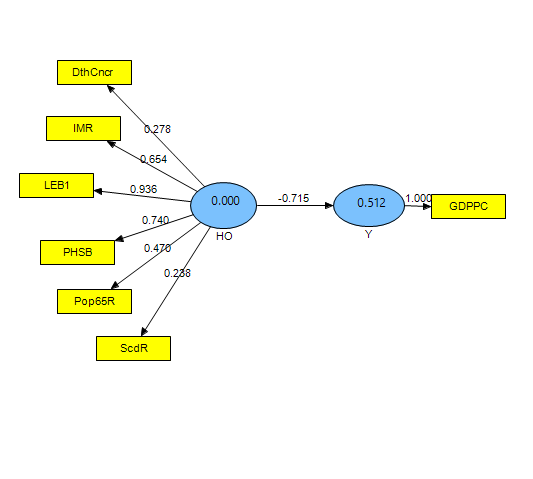 | 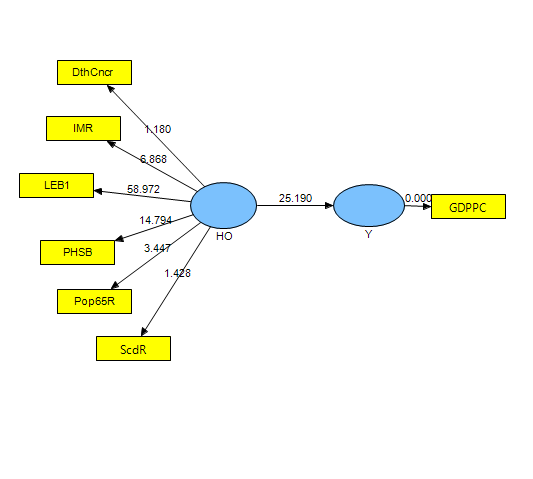 |
| 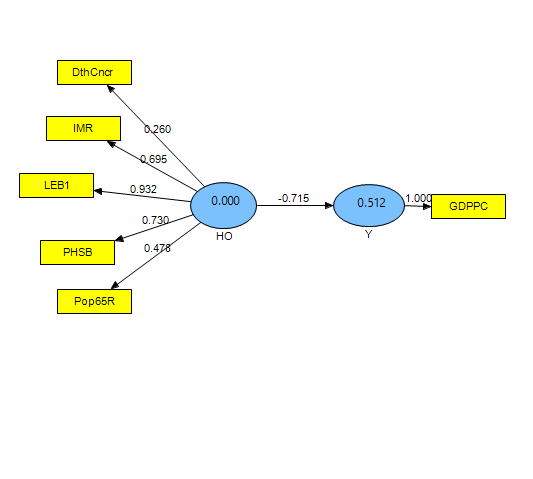 | 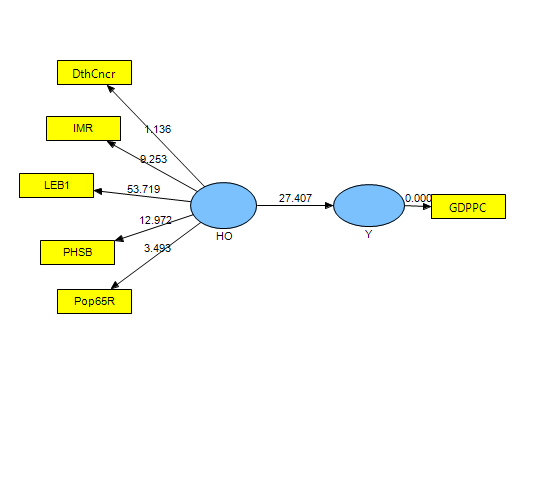 |
| 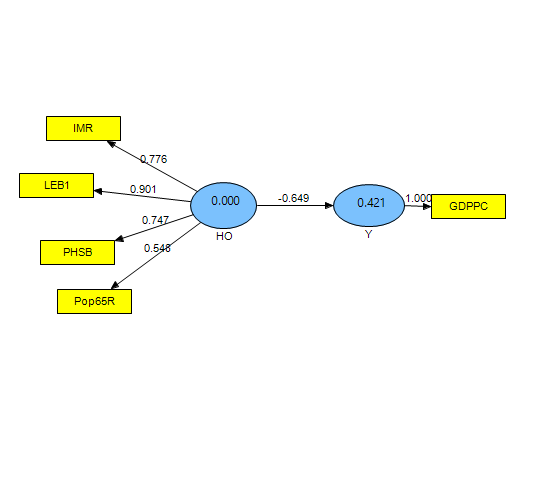 | 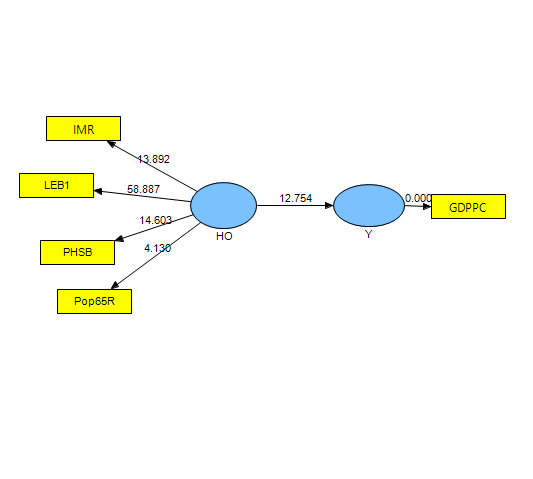 |

Note: See Appendix A for the abbreviations of the variables. 0.512 and 0.421 are R-Sq values indicating the strength of the relationship. *T*-values indicate the significance of the relationship. DthCncr = number of death due to cancer, Pop65R = population of over 65 years and above as percentage of total population and HO = health outcomes.

For all the estimated models in figure 2, IMR, LEB1 and PHSB remained significant (based on the estimated *T*-values) irrespective of the inclusion and exclusion of several key indicators of health outcome. This study used an inclusion value of 0.6 for the factor loadings. Which means items with loading value more than 0.6 were included in the model [81]. Hence, the final model used these three variables for constructing the health outcome index.

**Table 6: The calculated health outcome index with equal weights for 2014**

| **Ranking** | **Country** | **HO*index*** | **Ranking** | **Country** | **HO*index*** |
| --- | --- | --- | --- | --- | --- |
| 1 | Sweden | 0.923591 | 16 | Belgium | 0.737301 |
| 2 | Switzerland | 0.901994 | 17 | Denmark | 0.732333 |
| 3 | Iceland | 0.900172 | 18 | United Kingdom | 0.723817 |
| 4 | Ireland | 0.851559 | 19 | Greece | 0.696258 |
| 5 | Norway | 0.851086 | 20 | United States | 0.693065 |
| 6 | Spain | 0.846319 | 21 | Israel | 0.643151 |
| 7 | Canada | 0.845491 | 22 | Korea | 0.638183 |
| 8 | Finland | 0.835828 | 23 | Czech Republic | 0.630022 |
| 9 | Netherlands | 0.825774 | 24 | Portugal | 0.553968 |
| 10 | Luxembourg | 0.810753 | 25 | Poland | 0.477441 |
| 11 | France | 0.802118 | 26 | Estonia | 0.452720 |
| 12 | Austria | 0.765215 | 27 | Slovak Republic | 0.412624 |
| 13 | Italy | 0.764978 | 28 | Hungary | 0.353957 |
| 14 | Germany | 0.762613 | 29 | Latvia | 0.294581 |
| 15 | Slovenia | 0.748419 | 30 | Lithuania | 0.288667 |
|  |  |  | 31 | Turkey | 0.284054 |

Note: For all the indicators equal weights (0.33) has been used.

Table 6 represents the HO*index* constructed with assigning equal weights (instead of the calculated weights in the original model), to estimate the sensitivity of the index to weights. Therefore, the new equation for estimating the index is,

HO*index* = ∑ (ZLEB1 x W*_EQ_ +* ZIMR x W*_EQ_ +* ZPHSB x W*_EQ_*)

here W*_EQ_* = 0.33. The ranking of countries and HO*index* values in Table 1 and Table 6 are approximately identical. Which is an indication that the constructed HO*index* is not sensitive to weights change.

**5. The stationarity of the variables and their long-run relationship**

**Table 7: Panel unit root and panel cointegration tests**

| **Variables** | **IPS unit root test at level (Trend)** | **ARDL (long-run)** |
| --- | --- | --- |
| ICT*index* | -2.94* | -0.005(0.002)* |
| Gini | -5.67* | 0.006 (0.001)* |
| RdExp | -1.53* | 0.303 (0.08)* |
| ObstM | -1.98* | -0.034(0.008)* |
| lnGDPpc | -0.42 | 0.057 (0.026)* |
| lnHrWk | -1.67* | -0.183 (0.203) |
| SucdRt | -1.67* | -0.043(0.014)* |
| TerEdu | -3.48* | 0.0001 (0.000) |
| HO*index* | -1.73* |  |

Note: * Significant at 5% level. The lag order is chosen using AIC criterion. Figures in brackets are the standard errors. IPS indicates Im-Pesaran-Shin panel unit root test. The IPS test has Ho = All panels contain unit root. The ‘demean’ option was used to account for the cross-section dependence in the panel data [82]. Akaike’s Information Criterion (AIC) was used for lag selection. ARDL stands for autoregressive distributed lag model. Only long-run relationships are demonstrated. All variables are stationary at level except for lnGDPpc, which is stationary at the 1^st^ difference.

Table 7 shows the panel unit root test and panel cointegration test for the variables of interest in this study. The IPS test proposed by Im et al. [83] is suitable for a dynamic heterogeneous panel. On the other hand, the ARDL model is consistent in estimating the long-run association regardless of whether the regressors are stationary at level or at 1^st^ difference [84]. The results of the tests indicate the panel data do not contain unit roots and except for tertiary education and log hours of work per person per year, all other variables have a long-run relationship with health.
